# Real-time PCR on skin biopsies for super-spreaders’ detection in bovine besnoitiosis

**DOI:** 10.1186/s13071-020-04405-7

**Published:** 2020-10-22

**Authors:** Christelle Grisez, Leslie Bottari, Françoise Prévot, Jean-Pierre Alzieu, Emmanuel Liénard, Fabien Corbière, Marie Rameil, Xavier Desclaux, Christophe Lacz, Christian Boulon, Julie Petermann, Jeanne Le Mével, Carine Vilardell, Philippe Jacquiet

**Affiliations:** 1 UMR INRA/DGER 1225, Ecole Nationale Vétérinaire de Toulouse, 23 chemin des Capelles, 31076 Toulouse Cedex 03, France; 2 LVD 09, Laboratoire Départemental de l’Ariège, rue de Las Escoumes, 09008 Foix CDIS, France; 3 UMR INTHERES/DGER, Ecole Nationale Vétérinaire de Toulouse, 23 chemin des Capelles, 31076 Toulouse Cedex 3, France; 4 FRGDS Occitanie, 96, rue des agriculteurs - BP 102, 81003 Albi Cedex, France; 5 GDS Ardèche, 4 Avenue de l’Europe Unie, 07000 Privas, France; 6 GDMA Indre, 4 Rue Robert Mallet-Stevens, 36000 Châteauroux, France; 7 GDS des Alpes de Haute Provence, 66 Boulevard Gassendi, 04000 Digne-les-Bains, France

**Keywords:** *Besnoitia besnoiti*, Cattle, Control, Super-spreaders, Real-time PCR

## Abstract

**Background:**

Bovine besnoitiosis, an emerging disease in Europe that can be transmitted by vectors, is caused by the apicomplexan *Besnoitia besnoiti*. Bovine besnoitiosis is difficult to control due to the complexity of its diagnosis in the acute stage of the disease, poor treatment success and chronically asymptomatic cattle acting as parasite reservoirs. When serological prevalence is low, detection and specific culling of seropositive cattle is feasible; however, economic considerations preclude this approach when serological prevalence is high. The aims of this study were to evaluate the accuracy of detection of super-spreaders in highly infected herds and to test their selective elimination as a new control strategy for bovine besnoitiosis.

**Methods:**

Previous real-time PCR analyses performed on skin tissues from 160 asymptomatic animals sampled at slaughterhouses showed that the tail base was the best location to evaluate the dermal parasite DNA load. All seropositive animals (*n* = 518) from eight dairy or beef cattle farms facing a high serological prevalence of besnoitiosis were sampled at the tail base and their skin sample analysed by real-time PCR. A recommendation of rapid and selective culling of super-spreaders was formulated and provided to the cattle breeders. Subsequent serological monitoring of naïve animals was used to evaluate the interest of this control strategy over time.

**Results:**

Among the 518 seropositive animals, a low proportion of individuals (14.5%) showed Cq values below 36, 17.8% had doubtful results (36 < Cq ≤ 40) and 67.8% had negative PCR results. These proportions were grossly similar on the eight farms, regardless of their production type (beef or dairy cattle), size, geographical location or history of besnoitiosis. Within two weeks of the biopsy, the rapid culling of super-spreaders was implemented on only three farms. The numbers of newly infected animals were lower on these farms compared to those where super-spreaders were maintained in the herd.

**Conclusions:**

Real-time PCR analyses performed on skin biopsies of seropositive cattle showed huge individual variabilities in parasite DNA load. The rapid culling of individuals considered as super-spreaders seems to be a new and encouraging strategy for bovine besnoitiosis control.
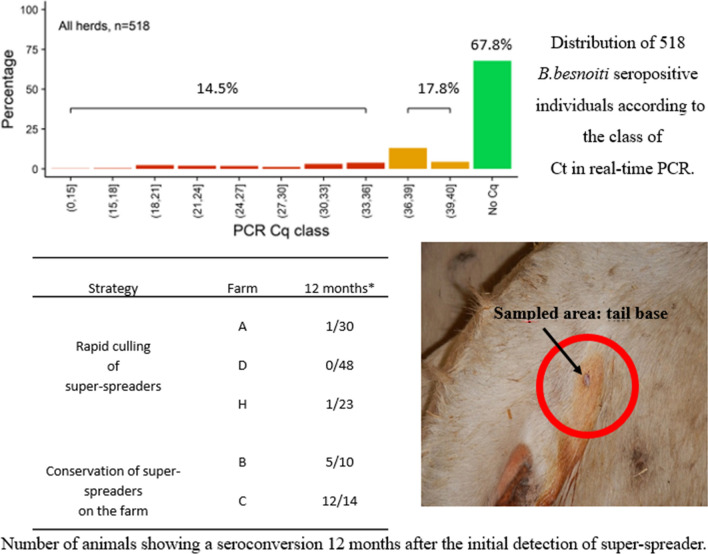

## Background

Bovine besnoitiosis is a re-emerging protozoan disease in Europe caused by the cyst-forming apicomplexan parasite *Besnoitia besnoiti* [1, 2]. The tachyzoite and bradyzoite stages of the parasite develop in the cattle host and are respectively responsible for the acute and chronic phases of this disease. During the acute stage of the disease, tachyzoites multiply quickly within bovine macrophages and endothelial cells of blood vessels [3]. The subsequent chronic phase, which can last several years, is characterized by massive skin alterations resulting from the formation of numerous cysts containing thousands of bradyzoites. This chronic and debilitating disease is responsible for heavy economic losses in cattle, and sometimes death. However, in most infected and seropositive cattle, no obvious clinical signs are observed.

The transmission routes of *B. besnoiti* have long been the subject of debate. No definitive hosts have been identified in Europe [4], no parasite DNA has been detected in semen of *B. besnoiti* infected bulls [5] and transplacental or colostral transmission is unlikely [6]. The presence of parasite cysts in the skin suggests that the main mode of transmission is likely mechanical, with hematophagous flies transmitting the parasite as experimentally demonstrated by Bigalke [7] and Sharif et al. [8]. Bigalke [7] regarded chronic cases as being of greater importance as sources for susceptible cattle than acute cases and Sharif et al. [8], demonstrated that a low number of stable flies (*n* = 300) were able to transmit bradyzoites from a chronically infected cow to a susceptible recipient host. The experiments of Bigalke [7] have clearly shown that clinically and even some asymptomatic long-lasting infected cases of besnoitiosis act as very efficient sources of infection for susceptible animals.

It is difficult to control bovine besnoitiosis. Life vaccines are available in some countries [9] but are not registered in Europe and need further improvement before a large use in the field [3]. Sulfonamides have been proposed to diminish the clinical signs during the acute stage of the disease [10] but are totally ineffective in the chronic phase when the parasite is protected within the cysts. Resistance to insecticides has been increasingly detected in the field in France [11] and Germany [12], making the control of vector transmission highly hypothetical. Finally, the control of bovine besnoitiosis relies entirely on flock management measures implemented after diagnostic test results are known. Biosecurity and biocontainment management measures, such as the rigorous serological testing of new animals prior to entering a herd, is a key point. In an infected herd, the seroprevalence should be evaluated rapidly after the initial diagnosis [13]. Severe clinical cases must be systematically and rapidly eliminated from the herd; such animals only represent a small proportion of seropositive animals in both endemic and epidemic areas. Recommendations differ sharply depending on the intra-herd seroprevalence level. When the seroprevalence is low, i.e. below 10%, the rapid and selective culling of seropositive animals is considered as the best option to avoid the spread of the disease within the herd. However, in herds with high seroprevalence, very few options remain available because the rapid elimination of the entire group of seropositive animals is not possible for economic reasons. Keeping seropositive animals separate from non-infected ones until they are gradually replaced by healthy and seronegative animals is a feasible and successful approach [14]. However, not all farms can manage two separate herds over several years. This is especially true for dairy cattle farms, where non-infected and infected animals are brought together during milking. In this context, the rapid detection and culling of super-spreaders identified by Bigalke [7] could be a realistic option for bovine besnoitiosis control. However, the proportion of asymptomatic infected animals which show high numbers of parasite cysts in their dermis compared to the total number of infected and seropositive animals is not known. Cysts in the dermis could be detected using histology and immunohistochemistry [15], but this method is time-consuming and probably lacks sensitivity. Cortes et al. [16] described a sensitive and specific real-time ITS1 rDNA PCR test which allows the detection of *B. besnoiti* infections in bovine skin biopsies. The sensitivity of the amplification reaction was high in that this test allowed the detection of DNA equivalent to one *B. besnoiti* cell. In this study, an immunofluorescence antibody test, histopathological analysis and real-time PCR were performed on 20 asymptomatic animals. Four groups of animals were identified: (i) 12 animals showing negative results in an indirect fluorescent antibody test (IFAT), histology and PCR; (ii) three animals with positive results for the three tests; (iii) three animals with positive IFAT and PCR results and negative histology results; and (iv) two animals with positive IFAT results and negative histology and PCR results. These results suggest that parasite DNA could be detected in the skin of some asymptomatic and infected animals and not in others. Therefore, real-time PCR on skin biopsies could identify super-spreaders, i.e. cattle whose dermis contains a high level of parasite DNA load. Nevertheless, it is a prerequisite to know the optimal sites for sampling skin biopsies, i.e. with the highest probability of finding tissue cysts and parasite DNA. In a study performed on a very low number of chronically and clinically infected cattle, Schares et al. [17] demonstrated that *B. besnoiti* cysts were not equally distributed in the skin, with the highest parasite DNA concentrations in the rump and in the distal parts of hind legs, and the lowest parasite DNA concentrations in the ventral, head and neck regions of the body. The reasons for this heterogeneous distribution of cysts are so far unknown.

This study aimed to (i) assess the distribution of parasite DNA in the severe chronic stage of the disease, (ii) compare the parasite DNA concentrations between three skin locations of asymptomatic infected cattle (base of the tail, neck and ear), (iii) evaluate the proportion of individuals showing high parasite DNA concentrations in their skin in herds with high seroprevalence, and (iv) assess the feasibility and the efficacy of a new control strategy for bovine besnoitiosis based on the rapid detection and culling of individuals considered to be super-spreaders.

## Methods

### Study 1: Distribution of parasite DNA in the severe chronic stage of the disease

Fourteen adult (from 2 to 12 years-old) cattle (11 females and 3 males) of various breeds, including Salers, Limousine, Charolaise, Gasconne des Pyrénées and Blonde d’Aquitaine, showing advanced clinical signs of the chronic phase of the disease were sent to the National Veterinary School of Toulouse by cattle breeders located within a 200 km radius around Toulouse. Before euthanasia, to confirm the diagnosis of besnoitiosis, blood samples for western blot (WB) analyses were collected in BD Vacutainer® (Dutscher, Brumath, France) blood collection tubes without anticoagulants. These animals were humanly euthanized by a veterinary surgeon using a lethal intra-venous injection of embutramide (T61, Intervet) and immediately necropsied.

For each animal, skin samples (1 cm^2^) were collected immediately after death from several sites: right foreleg; right hind leg; udder for females; right inner thigh; backline; right flank; right shoulder; right eyelid; dewlap; umbilicus area; tail base; and ocular sclera for external zones. Tissue samples (1 cm^2^ or 1 cm^3^) from internal organs, lung (lower respiratory tract), spleen, liver, heart, right kidney, diaphragm, subcutaneous connective tissue, cutaneous muscle collected from right flank, nasal and tracheal mucosa (upper respiratory tract)) also were collected. All samples were transported individually in an identified dry tube (Corning MCT-150-G; Fisher Scientific, Illkirch, France) and stored at 4 °C before DNA extraction and PCR analysis carried out the following day.

#### DNA extraction and quantitative real-time PCR

In the ENVT (Ecole Nationale Vétérinaire Toulouse) laboratory, DNA was extracted from tissue biopsies using a commercial kit (QIAmp® DNA Mini Kit; Qiagen, Courtaboeuf, France). Following the manufacturer’s recommendations, 50 mg aliquots of tissue biopsies were processed after an over-night incubation with proteinase K. *Besnoitia* spp. internal transcribed spacer 1 (ITS1) amplification was performed with the commercial PCR kit AdiaVetTM Besnoitia (AES Chemunex, Bruz, France). The quantitative PCR was performed with the Stratagene MX3005P thermal cycler (Agilent Technologies, La Jolla, CA, USA). Positive and negative controls were provided by the manufacturer. Results were analyzed using the MxPro QPCR version 4.10 software (Agilent Technologies). When the Cq value was inferior or equal to 36, the parasite DNA was considered detected and the animal was deemed to be a super-spreader, when Cq values ranged between 36 and 40, parasite DNA was at the limit of detection, and a Cq superior to 40 was considered as a negative real-time PCR result.

#### Western blot analyses

WB analyses were performed on animals necropsied in the ENVT facilities, on animals collected in the slaughterhouses, and when doubtful ELISA results were recorded on farms A to H. For tachyzoite-based WB analysis, the coated membranes and the immunoblots were performed as previously described [18]. Three main antigenic reactivity areas are described [19]: area I, 12–20 kDa; area II, 23–38 kDa; and area III, 60–90 kDa. The minimal criterion for serological positivity was the recognition of at least four bands in at least two domains. This test is considered to be highly specific [20].

### Study 2: Comparison of the DNA concentration between three skin locations in asymptomatic infected cattle

One hundred and sixty adult cattle were sampled in three slaughterhouses located in endemic areas: Ariège (*n* = 105), Hautes-Alpes (*n* = 33) and Alpes de Haute-Provence (*n* = 22). These animals did not show any clinical sign of bovine besnoitiosis and their status (infected or not) was unknown at the time of sampling. Skin biopsies for PCR were collected from the ear, neck and tail base, transported individually in an identified dry tube (Corning MCT-150-G) and stored at 4 °C until PCR analysis carried out the following day as described in Study 1. Blood samples for WB analyses were drawn from the jugular vein during bleeding and collected in BD Vacutainer® blood collection tubes without anticoagulants. WB analyses were processed as described in Study 1.

### Study 3: Evaluation of the DNA concentrations in skin samples from herds with high seroprevalence

Eight farms located in different regions of France (2 in the center, 6 in the south) were included in the study (Table 1). Both dairy (*n* = 4) and beef cattle herds (*n* = 4) were selected. Farms A, B and C were recruited in the spring of 2017, farms D, E and F in the spring of 2018 and farms G and H in the spring of 2019. These farms were chosen because (i) all had experienced at least one clinical case of bovine besnoitiosis before the beginning of the survey, (ii) the enzyme-linked immunosorbent assay (ELISA)-seroprevalence established in the two weeks before the beginning of the survey was high (over 40%), and (iii) the cattle producer volunteered to participate in the study. All seropositive individuals (*n* = 518) of these 8 farms were tested by real-time PCR on skin biopsies. Detailed information is provided in Table 1.

**Table 1:** Description of the eight studied farms

Farm	Husbandry	Breed	No. of individuals examined	Initial seroprevalence (%)^a^	No. of clinical cases (period)
A (Aveyron)	Dairy cattle	Prim’Holstein	32	42	2 (2016)
B (Tarn)	Beef cattle	Blonde d’Aquitaine	49	67 (> 2 years); 50 (< 2 years)	6 (2014–2016)
C (Ariège)	Beef cattle	Limousine	32	92	3 (2015–2017)
D (Ardèche)	Beef cattle	Charolaise	34	51	nd
E (Cher)	Dairy cattle	Prim’Holstein	161	74	nd
F (Indre)	Beef cattle	Charolaise	163	80	6 (2016–2018)
G (Ardèche)	Dairy cattle	Montbéliarde	25	52	nd
H (Ardèche)	Dairy cattle	Montbéliarde	22	88	4 (2018)

^a^In the weeks before PCR-skin sample analysis

*Abbreviation*: nd, not documented

According to the results of Study 2, the skin sample for PCR was taken without anesthesia at the tail base using biopsy punches (8 mm diameter; Kruuse, Langeskov, Denmark). This area was previously cleaned with Betadine^®^ (Centravet, Castelnaudary, France). The skin fragment was placed in a dry tube (Corning MCT-150-G), identified with the bovine individual ID, stored at +4 °C and analyzed within two days as described in Study 1. Aluminum spray (Aluspray® Vetoquinol; Centravet, Castelnaudary, France) was sprayed on the biopsied area.

#### ELISA analyses

Sera were separated by centrifugation and tested for *B. besnoiti* antibodies using a commercial ELISA kit (ID Screen® Besnoitia Indirect 2.0; IDVET, Grabels, France). Serological analyses were performed by the Departmental Veterinary Laboratory of Ariège (LVD 09) for farms A, B, C and D, LVD 26 (Drôme) for farms G and H, LVD 18 (Cher) for farm E, and LVD 36 (Indre) for farm F.

### Study 4: Efficacy of the culling of super-spreaders in the control of bovine besnoitiosis

Real-time PCR analyses were performed within two days after the skin biopsy samples were collected. The results were immediately communicated to farmers and veterinarian practitioners in charge of the farm so that animals deemed to be super-spreaders (Cq ≤ 36) could be culled as soon as possible. In this way, super-spreaders were discarded from the herd before the high activity period of hematophagous flies began. However, this culling strategy was only applied on farms A, D and H. To assess whether the status of individuals persisted over time, PCR samples were collected again 10 months later on 6 animals considered to be super-spreaders on farm C, and 7 animals with doubtful or negative PCR results were sampled three years later on farm A. All real-time analyses were performed as described in Study 1.

To assess the ability of this strategy to reduce the number of newly acquired *B. besnoiti* infections, serological ELISA analyses were performed on previously seronegative animals and young ones (over 6 months-old) that were not present on the farm at the previous sampling date. The time intervals between the initial and subsequent analyses differed per farm as follows: 12, 24 and 36 months for farm A; 12 and 24 months for farm D; 12 months only for farms B, C and H. ELISA analyses were performed as described in Study 3.

### Statistical analyses

In chronically infected cattle necropsied in the ENVT facilities, comparisons between mean Cq values from skin samples and mean Cq values from internal organs, and then between mean Cq values from skin of the upper side of the body and from skin of the lower side of the body, were performed using Mann-Whitney-Wilcoxon (MWW) rank tests. Individual variability of Cq values obtained in asymptomatic infected animals from the 8 farms studied were presented according to classes of Cq (in steps of 3 Cq units between Cq ≤ 15 to no Cq). Distribution of individuals within these Cq classes was compared between farms using the Fisherʼs exact Chi-square test. A binary logistic regression model was fitted to investigate factors (farm, age and ELISA S/P value) related to the Cq value obtained in real-time PCR on the skin biopsies (positive for Cq value < 40 *versus* negative for Cq > or equal to 40). These factors included farm (A, B, C or D), age (below or above 24 months-old) and ELISA S/P value (below and above 110%). Only the 147 individuals from farms A, B, C and D were used in this analysis because these analyses were performed in the same laboratory (Laboratoire Vétérinaire Départemental de l’Ariège). All statistical analyses were done using R software (version 3.5.2, R foundation; www.r-project.org).

## Results

### Study 1: Distribution of parasite DNA in the severe chronic stage of the disease

**Fig. 1:**
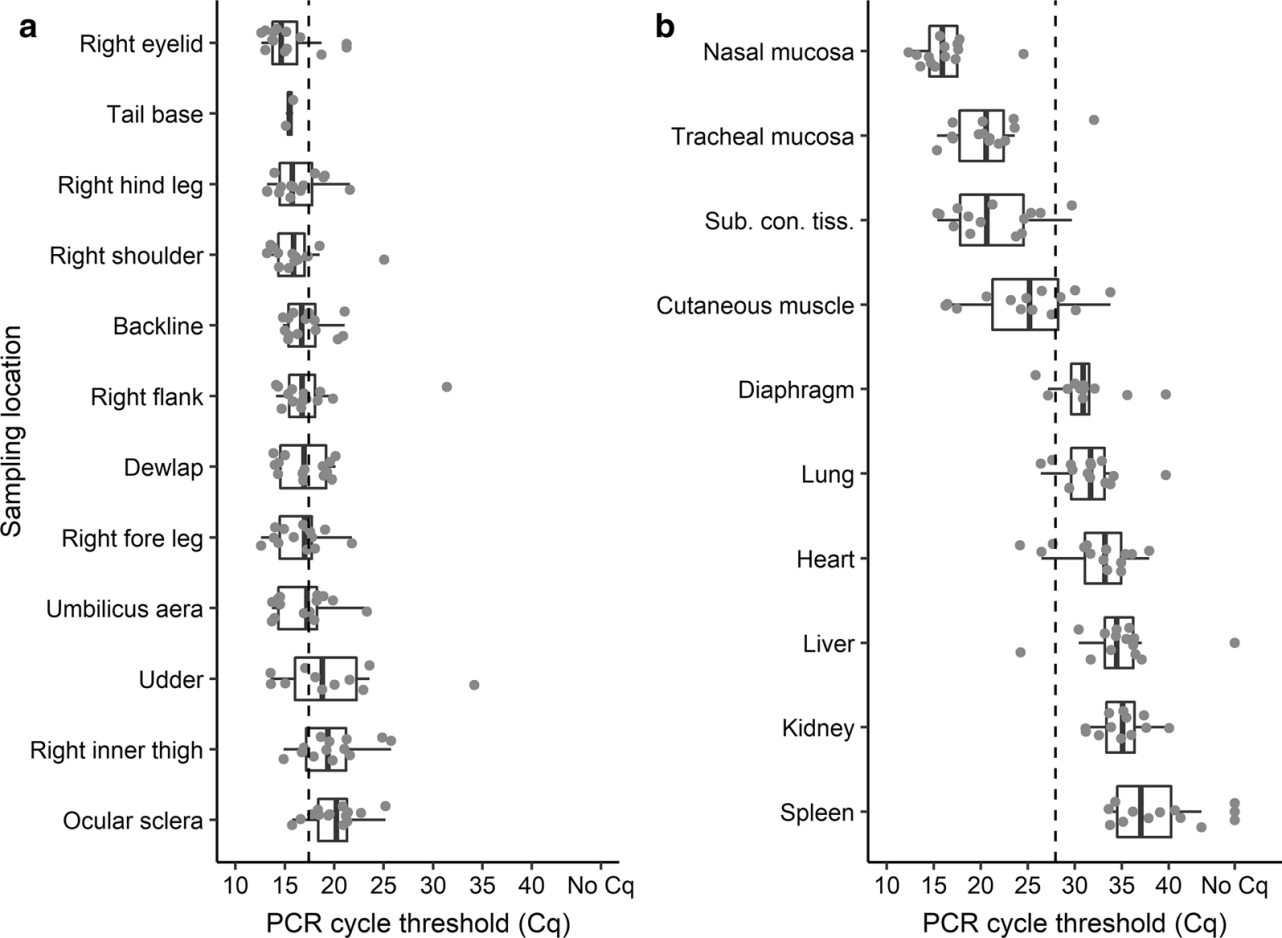
Real-time PCR results (expressed as mean Cq values and SD) of 14 *B. besnoiti* infected cattle showing severe scleroderma, slaughtered in the facilities of the National Veterinary School of Toulouse. **a** Skin samples. **b** Internal organs

All tissues from the 14 animals in the severe chronic phase of the disease yielded real-time PCR positive results (Fig. 1a, b). A significantly higher DNA parasite load (Wilcoxon signed rank test, *W* = 2704, *P* < 0.0001) was found in skin samples (median Cq value: 17.0; range: 12.6–34.2) compared to internal organs (median Cq value: 30.0; range: 12.3–39.7). Mean Cq values in skin samples ranged from 15.0 (tail base) to 20.0 (udder) but no significant difference was observed between the lower body parts (right anterior and posterior barrels, udder, interior of the right thigh, dewlap and umbilical zone) and the upper body parts (back line, right flank and right shoulder). The parasite DNA load was higher (Wilcoxon signed rank test, W = 105, *P* = 0.0002) in the upper respiratory tract than in the lower respiratory tract (lung).

### Study 2: Comparison of the DNA concentration between three skin locations in asymptomatic infected cattle

Out of 160 animals sampled at three slaughterhouses, 113 (70.6%) yielded negative WB results (Table 2). Among them, no parasite DNA was found in 97 individuals (85.8%) whatever the sampled area (tail base, ear or neck). Nevertheless, parasite DNA was detected in low amounts in 14 animals (12.4%) in at least one location and in large amounts in only 2 animals (1.8%). Forty-seven animals (29.4%) exhibited a positive WB analysis. No parasite DNA was detected in half of them (24 animals) whatever the location of the skin sample. Parasite DNA was detected in the 23 remaining individuals, 8 with low parasite loads only and 15 with at least one high parasite DNA load. Among the 23 animals showing positive parasite DNA detection, 7 had only one positive location, 12 had two positive sampling sites and only 4 had three positive locations. Regarding the WB-positive individuals showing at least a Cq value below or equal to 36 in the qPCR (*n* = 17), the tail base was positive in 12 cases (70%), and neck and ear samples were positive in only 7 animals (41%).

**Table 2:** Distribution of 160 necropsied cattle according to PCR Cq values and western blot (WB) results (slaughterhouses in southern France, endemic areas of besnoitiosis)

WB	PCR results			Total
	Cq ≤ 36	36 < Cq ≤ 40	No Cq	
Positive	15	8	24	47
Negative	2	14	97	113
Total	17	22	121	160

### Study 3: Evaluation of the DNA concentrations in skin samples from herds with high seroprevalence

Out of the total number of cattle examined (*n* = 518), 75 (14.5%) yielded a highly positive real-time PCR result (Cq ≤ 36) on skin samples taken from the tail base, 92 (17.8%) were “doubtful” with a parasite DNA amount close to the limit of detection (36 < Cq ≤ 40), and 351 individuals (67.8% of the total) were “negative”, i.e. no Cq.

**Fig. 2:**
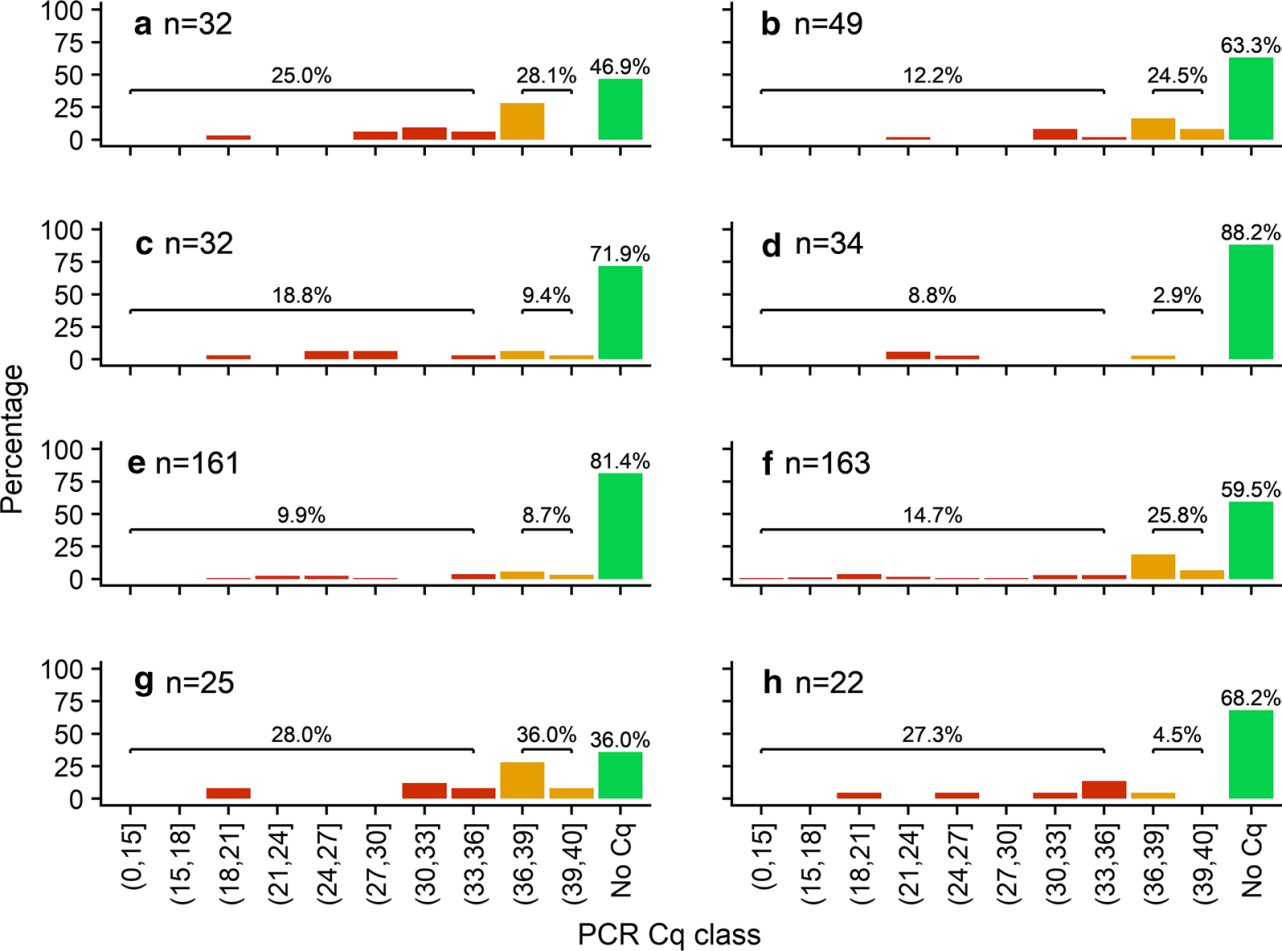
Distribution of *B. besnoiti* seropositive individuals according to the class of Cq in real-time PCR on the eight farms (detailed results)

This general trend of the distribution of individuals into three main categories of Cq values was similar in the eight farms studied, with the category “no Cq” predominating, i.e. no parasite DNA detected, regardless of the animals’ geographic origin, size, type of production (dairy *versus* beef cattle farms) or history of the disease. However, some variations in the balance between the three categories were noted according to the farm (Fisher’s exact test, *P* = 0.0005) (Fig. 2). The potential influence of some factors (farm, age and optical densities in ELISA) on the PCR status of an individual (positive when Cq value < 40 *versus* negative for Cq > or equal to 40) were analyzed by fitting multivariable logistic regression models (Table 3). All investigated factors had a significant effect on the binary positive/negative PCR response. In this study, animals below 24 months-old and animals with high ELISA optical densities (over 110%) had significantly higher odds of having a positive real-time PCR response.

**Table 3:** Results from the multivariable logistic regression model fitted to the binary positive/negative real-time PCR response for 147 cattle

Variable	Cq < 40^a^	Cq ≥ 40^a^	OR	95% CI	*P*-value
Farm A	17	15	Ref		
Farm B	18	31	0.36	0.13–0.94	392.10^−2^
Farm C	9	23	0.12	0.03–0.43	1.86.10^−3^
Farm D	4	30	0.01	0.00–0.07	5.21.10^−6^
Age ≤ 24 months	34	65	Ref		
Age > 24 months	14	34	0.09	0.02–0.38	1.85.10^−3^
OD < 110%	16	55	Ref		
OD ≥ 110%	32	44	2.60	1.18–5.93	1.98.10^−2^

^a^Number of individuals

Abbreviations: OR, odds ratio; CI, confidence interval; OD, ptical density in ELISA; Ref, reference

### Study 4: Efficacy of the culling of super-spreaders in the control of bovine besnoitiosis

After receiving the results, two types of strategies were followed. Farmers A, D and H decided to cull or to separate in a confined place the animals deemed to be super-spreaders (i.e. with Cq values below or equal to 36) in the week following the reception of the results. In contrast, farmers B and C decided to keep the super-spreaders in their herds, either because these animals were of high genetic merit (farm B) or because they were young replacement heifers (farm C). The subsequent course of besnoitiosis infection strongly differed between these two groups of farms (Table 4). Seven seropositive cattle from farm A showing initial PCR results that were negative or questionable (36 < Cq ≤ 40) were tested again 36 months later and similar results were obtained. This suggests that the status of non “super-spreaders” could be stable over time (Table 5). Similarly, 6 seropositive animals considered to be super-spreaders from farm C were tested again 10 months later; the status of these animals had not changed.

**Table 4:** Number of animals showing a seroconversion and total number of animals examined, in the following months after the initial detection of super-spreaders, according to the strategy used by farmers

Strategy	Farm	Time after the initial detection of super-spreaders				
		6 months	12 months	18 months	24 months	36 months
Rapid culling of super-spreaders	A		1/30	2/30	3/54	0/61
	D		0/48		6/14	
	H		1/23			
Conservation of super-spreaders in the farm	B	15/30	5/10			
	C		12/14

**Table 5:** Real-time PCR results obtained on skin biopsies 12 months (farm C) or 36 months (farm A) after the initial evaluation

Farm	Animal	First real-time PCR result (Cq value)	Second real-time PCR results (Cq value)	
			10 months later	36 months later
A	1	38		No Cq
	2	38		38
	3	39		No Cq
	4	No Cq		No Cq
	5	No Cq		No Cq
	6	37		No Cq
	7	No Cq		No Cq
C	1	34	35	
	2	26	26	
	3	21	24	
	4	25	16	
	5	29	26	
	6	29	36	

## Discussion

When the serological prevalence is below 10% in a cattle herd, the rapid detection and subsequent culling or separation of all seropositive individuals is currently the most effective way to prevent the within-herd spread of besnoitiosis [14]. For seroprevalence above 10% or due to specific conditions on a farm, this sanitation strategy is not practically or economically feasible. In this context, this work was carried out to propose a selective culling strategy to cattle breeders who have herds with a high seroprevalence and are unable to manage two separate batches of animals. Current observations agree that all seropositive cattle on a farm do not contribute equally to the transmission of *B. besnoiti*. Individuals showing clinical signs of a chronic disease have an enriched-cysts dermis [21], and their culling should reduce the transmission pressure within the herd. However, this measure cannot totally control besnoitiosis in a herd because a subset of seropositive and asymptomatic animals may also present a risk of parasite transmission to seronegative animals on the farm [22]. Indeed, among seropositive cattle that have never shown any obvious clinical sign of the disease (febrile phase and/or scleroderma), it is reasonable to consider that some of them may carry a significant number of cysts in their tegument [23]. This category of animals could serve as a reservoir of parasites and could play a major role in the transmission of the parasite *via* hematophagous flies. According to this assumption, the identification and selective culling of cattle that have high concentrations of bradyzoite cysts in their skin, even if they do not exhibit clinical signs, could be a major tool to control the disease in endemic areas. Previous studies carried out on a small number of cattle have shown that the PCR method allowed to detect *B. besnoiti* DNA in skin samples collected from seropositive cattle with no clinical signs of a chronic disease [16].

In the present survey, the use of a real-time PCR analysis on a skin sample made it possible to distinguish between individuals with a high parasite DNA load and those with a low or no parasite DNA load. In the severe chronic phase with elephant skin, bradyzoite cysts are easily detected in the dermis, the scleral conjunctiva and mucous membranes of the upper respiratory tract by histology [24] and real-time PCR [16, 25]. This feature was also observed in samples taken from the skin and organs of the 14 chronically infected cattle of the present study: the skin samples showed significantly higher parasite DNA loads than the internal organs, except for the nasal mucosa whose mean Cq value was very low. The skin is the site of predilection for cysts, and considering the results obtained in cattle showing scleroderma, all skin biopsies, regardless of the site sampled, yielded Cq values below 34.2. Schares et al. [17] collected samples from 77 different skin locations in four chronically infected cattle and observed a significant difference in the distribution and detectability of parasite DNA between skin regions. *Besnoitia besnoiti* is not equally distributed in the skin of infected cattle. The tail base with an average Cq value of 15.5 ± 0.5 was particularly rich in parasite DNA and provided an easily accessible site for a biopsy to be carried out on a living animal on a farm. In contrast, PCR analyses revealed more heterogeneous results in asymptomatic infected animals [23, 26]. In 24 out of 47 seropositive cattle in the slaughterhouse study, molecular amplification did not yield any positive result regardless of the site from which the skin sample was taken, while these individuals were confirmed to be infected by WB serology. However, during the course of infection, an animal recently infected could be seropositive but could not have developed cysts. Other clinically affected animals may experience an apparent recovery even though they remain infected life-long, making parasite DNA detection more difficult [27]. Strong humoral and cell-mediated immune responses [28] could significantly reduce the dermal cysts load, which could explain the negative PCR results of some skin regions [15, 21]. These negative results cannot be attributed clearly in the PCR sample process because (i) the quality and the efficiency of the DNA extraction from skin tissue was verified by the amplification of bovine GAPDH, an internal control of extraction and amplification steps, and (ii) the limit of detection of the commercial PCR tool was equivalent to the DNA of 2.5 parasites per reaction [5]. Moreover, even though *B. besnoiti* may cross with *Neospora caninum* [29], the high specificity of WB analysis in the diagnosis of besnoitiosis excludes a false positive result for such a large number of animals [20].

Samples taken from three locations (tail base, neck and ear) on the same individual did not give similar qPCR results. From the 39 cattle that tested PCR-positive in at least one of the three areas sampled, it was not possible to determine one location that gave a consistent positive qPCR result. Therefore, contrary to animals showing severe scleroderma, *Besnoitia* cysts appear to be heterogeneously distributed in the skin of asymptomatic cattle. As it is not feasible in field conditions to take several skin biopsies at the same time from one asymptomatic animal, it is essential to sample the skin site with the highest probability of having a cyst. The tail base yielded a positive result in 70% of seropositive animals with at least one Cq value below or equal to 36, while the positive rate for neck and ear samples was below 50%. An optimal compromise between the requirement for maximum sensitivity and the feasibility of sampling in field conditions therefore would be to designate the tail base as the location to sample to assess the status of one animal. However, this does not mean that a negative PCR result on a skin fragment taken from the tail base can be used to “qualify” this animal as “not dangerous for transmission”.

The distribution of PCR Cq values was similar in the eight farms, with a minority of individuals having a high parasite DNA load, regardless of geographical origin, production system, breed or herd size. Among asymptomatic seropositive cattle, a small proportion carried a much larger quantity of parasite DNA than others, and these animals considered as super-spreaders are likely to be a source of contamination for their naïve congeners in the presence of the vectors. As noted by Bigalke [7], if mechanical *B. besnoiti* transmission by *Stomoxys calcitrans* is of significance in the epidemiology of cattle besnoitiosis, the lack of parasite persistence on the mouthparts of stable flies suggests that mechanical transmission may only occur during brief periods of time [30]. This is in contrast with many blood-sucking vectors which are able to transmit pathogens for the rest of their life, such as *Culicoides* vectors of bluetongue virus [31] or phlebotomine sand fly vectors of leishmaniasis [32]. Therefore, in bovine besnoitiosis, the long persistence of infectivity in a minority of subclinical carriers could contribute in a large extent to the spread of the parasite within the herd. In the present study, PCR analysis of skin biopsies performed on 518 cattle highlights the heterogeneity of this infectivity, revealing a limited number of potential super-spreaders on each of the eight farms studied. The long persistence of super-spreader status was confirmed on only one farm by a second PCR analysis ten months after the initial sample (Table 5). This heterogeneity is largely shaping the epidemiology and transmission of infectious diseases. It opens herein the prospect of a new means to control besnoitiosis by identifying and selectively culling these super-spreaders. Schares et al. [25] noted a positive correlation between antibody titer and the amount of parasite DNA detected by PCR in 43 symptomatic cattle from the same cattle herd. Similarly, Frey et al. [27] found the highest parasite DNA loads in cattle with the highest antibody levels. Unfortunately, this correlation was not strong enough to use antibody titers as a proxy of real-time PCR analysis to detect super-spreaders, and many animals showed high antibody titers while yielding negative PCR results. The influence of some factors such as farm, age and antibody titer on the parasite load in the skin of cattle were confirmed. However, the multivariate analysis did not allow the determination of a typical profile of a super-spreader individual.

Farmers were proposed to cull very quickly or, at least, to isolate highly contaminated animals from the rest of the herd to reduce the transmission pressure. The ultimate goal of such a control strategy is to protect the healthy herd from new infections and to progress step by step towards a disease-free herd. However, many difficulties were encountered. The motivation of farmers to apply the recommendations differed from farm to farm. When cattle considered at risk of transmission were eliminated or segregated as soon as possible (farms A and D), the serological incidence was relatively low in the following months and years, excepted 24 months after the initial detection in farm D where an unexpected high level of serological incidence was observed, probably due to the close vicinity of other highly infected herds. However, these results were very encouraging and showed the potential efficacy of this strategy in reducing the parasite transmission within a herd. In contrast, these recommendations were not followed on farms B and C, and serological incidences in the following months and years were very high. On farm C, we accurately monitored heifers with highly positive serology and PCR results at two sampling periods (May 2017 and March 2018). Although the number of animals examined was low, it seemed that the super-spreader status persisted over time. These findings reinforce the desirability of eliminating these individuals as quickly as possible. Nevertheless, given the small number of farms that were included in this study, these assumptions need to be confirmed in further studies.

## Conclusions

In *B. besnoiti*-infected cattle, the parasite DNA load is massive in the skin of animals suffering from severe scleroderma but seems to be more heterogeneous in the skin of asymptomatically infected individuals. A real time PCR analysis on skin biopsies from the tail base might provide more accurate information on the infectiousness status of seropositive asymptomatic bovines compared to other locations and allows for the detection of super-spreaders in a herd. The proportion of super-spreaders was low on all of the studied farms and the rapid culling of these animals gave encouraging results in the control of bovine besnoitiosis in herds exhibiting a high seroprevalence. However, due to the small number of farms included in the present study, these preliminary results need to be confirmed at a large scale.

## Data Availability

Data supporting the conclusions of this article are included within the article.

## Abbreviations

ENVT: Ecole Nationale Vétérinaire de Toulouse
EFSA: European Food and Safety Authority
LVD: Departmental Veterinary Laboratory
PCR: Polymerase chain reaction
IFAT: Indirect fluorescent antibody test
ELISA: Enzyme-linked immunosorbent assay
WB: Western blot
ITS rDNA: Internal transcribed spacer ribosomal DNA
SD: Standard deviation
Cq: Quantification cycle
